# Neonatal Immunity to *Bordetella pertussis* Infection and Current Prevention Strategies

**DOI:** 10.1155/2019/7134168

**Published:** 2019-02-10

**Authors:** Carolina Argondizo-Correia, Ana Kelly Sousa Rodrigues, Cyro Alves de Brito

**Affiliations:** ^1^Institute of Tropical Medicine, University of São Paulo, Dr. Enéas de Carvalho Aguiar Avenue 470 Jardim América, São Paulo, SP 05403-000, Brazil; ^2^Immunology Centre, Adolfo Lutz Institute, Dr. Arnaldo Avenue 351 Cerqueira César, São Paulo, SP 01246-000, Brazil

## Abstract

*Bordetella pertussis* is the bacterial agent of whooping cough, an infectious disease that is reemerging despite high vaccine coverage. Newborn children are the most affected, not only because they are too young to be vaccinated but also due to qualitative and quantitative differences in their immune system, which makes them more susceptible to infection and severe manifestations, leading to a higher mortality rate comparing to other groups. Until recently, prevention consisted of vaccinating children in the first year of life and the herd vaccination of people directly in touch with them, but the increase in cases demands more effective strategies that can overcome the developing immune response in early life and induce protection while children are most vulnerable.

## 1. Introduction

*Bordetella pertussis* is a Gram-negative coccobacillus that causes whooping cough, also known as pertussis, in humans [1]. Historical reports mention the disease as far back as the XIIth century [2], but pathogen isolation only occurred in the XXth century [3]. Since then, much has been learned about the pathogenesis and prevention of the disease, but infection is still a concern in several countries [4].

Respiratory infection is especially aggressive in young children, who are more likely to experience the classical manifestation of the disease [5], divided into three phases: the first phase is characterized by unspecific symptoms, such as coryza, fever, and occasional cough. After two weeks, the cough is aggravated and becomes constant and uncontrollable, followed by forced inspiration producing a whooping sound. Symptoms can decrease progressively into the convalescence phase; however, complications such as pneumonia are frequent and are responsible for over 90% of the deaths attributable to the disease in children younger than 3 years of age [6, 7].

Until 2003, 50 million cases and 300,000 deaths were estimated every year around the world, mostly in children younger than 5 years of age [8].

Between 2010 and 2014, however, a rise in cases has been seen worldwide. In the US, the incidence before the 1980s was 1 case for each 100,000 inhabitants; in 2012, the incidence increased to 9 : 100,000, with more than 42,000 cases [6]. In the UK, over 9,000 children younger than 3 years old were infected in 2011 [9], and in Brazil, there were 22.426 confirmed cases, mostly in children younger than 1 year of age; in São Paulo, the largest state in the country, the incidence increased from 2.20 per 100,000 in 2011 to 5.06 per 100,000 in 2014 [10]. Other countries such as Argentina, Chile, Canada, and Australia also reported an increase in the number of cases [11, 12].

Treatment with macrolide antibiotics can be effective in eliminating the pathogen if administered at the beginning of the symptoms; but as these antibiotics are unspecific and the disease is usually diagnosed due to the paroxysmal cough, treatment is often delayed, and by the time it is prescribed, the symptoms are already more severe, making prevention vital, especially for young children [13].

## 2. Immunopathogenesis of Pertussis

When the bacteria enter the human body, they adhere to the respiratory epithelium and produce a number of pathogenic toxins [4] to break natural barriers, such as *cilia* and mucus, to evade the innate immune system [14]. Then, bacteria can reach epithelial cells and replicate intracellularly [1], leading to the recruitment of different arms of the immune system [15–18].

Briefly, the regular immune response against pertussis infection recruits both innate and adaptive immune responses. After recognition of bacterial patterns by Toll-like receptors (TLRs), resident macrophages and neutrophils phagocytize and destroy bacteria at the infection site while dendritic cells (DCs) present and activate T CD4^+^ lymphocytes, which in turn mainly differentiate into IFN-*γ*-producing-T helper (Th) 1 cells. Natural killer (NK) cells can also be recruited to produce IFN-*γ* to help polarize T cells. These molecules are especially important for activating macrophages through the production of IFN-*γ* to destroy bacteria that can survive phagocytosis and escape into the cell cytoplasm [18].

Pertussis can, however, use toxins to stimulate DCs to produce IL-10, instead leading to the differentiation of T regulatory cells and a predominance of an anti-inflammatory response, which is more favourable to the survival of the bacteria in the host [18].

In addition, antibodies, especially IgG and IgA, may have a role in bacterial clearance, even though there have been no previously defined correlates of protection [1, 19]. Antibodies can act by neutralizing bacterial toxins or as opsonins to prevent cell infection [1, 19], and maternal anti-pertussis antibodies are transmitted via the placenta to the foetus, contributing to newborn protection [18]. Nevertheless, more studies show that Th1 and Th17 responses are more efficient in rapidly clearing the bacteria [17, 20–22].

## 3. Neonatal Immunity

In children, several qualitative and quantitative differences in the immune response contribute to the severity of disease [23]. For a long time, neonates were considered most susceptible to disease due to a deficient and immature immune system [24]; however, it is currently known that the newborn immune system is only less responsive than that of the adult due to the regulatory effects of foetal-maternal tolerance that are imposed during development in the uterus and that remain active soon after birth [25].

Healthy newborns express TLRs in a stable way similar to that expression manner in adults and are capable of enhancing the expression of TLR on mononuclear cells in case of bacterial sepsis [26]. However, cells such as neutrophils, monocytes, and macrophages have more difficulty in expressing costimulatory molecules such as CD80 and CD86, secreting cytokines such as tumor necrosis factor *α*, IL-12p70, and IFN-*γ* and responding to chemokines until approximately 2 years of age [27–29]. They also have less capacity for antigen processing and less major histocompatibility complex II molecules, which participate in the activation of naïve T CD4^+^ cells and the differentiation of these cells to the Th1 profile, leading to anergy [25, 27–29].

Neutrophils are present in a lower number in the bone marrow in neonates and are more immature compared to those in adults, having fewer preformed antimicrobial peptides and a lower capacity for Gram-negative bacterial elimination, justifying their higher susceptibility to infection and sepsis [27, 30]. NK cell counts are elevated in newborns' peripheral blood, but these cells secrete small amounts of IFN-*γ* [29]. Impaired innate immunity can be partly explained by the immunomodulation by CD71^+^ erythroid suppressor cells. These cells are present in high numbers in neonates and human cord blood and are capable of inhibiting innate response to *B. pertussis* infection in neonatal mice by the expression of arginase II [31]. Similarly, the presence of erythroid suppressor cells decreases TNF-*α* production and *B. pertussis* phagocytosis by human CD11b^+^ cells in vitro.

These differences in the innate immune response are reflected especially in the development and in the profile of the newborn's adaptive immune response to *B. pertussis* [32]. Alongside not having a fully developed anatomical microenvironment that is suited to the interaction between DCs and T and B lymphocytes (there is no defined demarcation between different lymphoid zones and T CD4^+^ zones in neonates [28]), neonate cells have a lower capacity to generate memory cells and Th1 effector responses, also due to the lower IL-12 production by DCs [25]. Thus, there is lower subsequent production of IL-12 and IFN-*γ* [33] and a lower CD40L expression [27], which also lead to a lower production of IgG, IgA, and IgE [28], making the bacterial clearance compromised.

However, the production of IL-10, IL-6, IL-23, and IL-1*β*, cytokines that contribute to Th17 cell polarization, is higher in neonates than in adults [25, 26]. Additionally, elevated IL-10 production in early life was shown to be predominant in *B. pertussis*-infection cases [25, 34, 35].

While there is less Th1 polarization, there is higher IL-4 detection in neonate cells, both in unstimulated and in stimulated cells, compared to that in adult cells. The Th2 locus is hypomethylated in neonates and methylated in adults, while transcription of the subunit p35 of IL-12p70 in DCs derived from monocytes, when stimulated with lipopolysaccharide, is limited by epigenetic regulation [25, 36]. This makes Th2 cells more predominant in neonates, even after vaccination. However, it is known that the bacillus Calmette-Guérin vaccine can induce Th1 cells [37], so perhaps Th2 predominance is not a characteristic that is biased towards the newborn period, but is derived from the difficulty in polarizing cells to the Th1 profile during this period [25].

Th2, Th17, and regulatory lymphocyte predominance, allied to the absence of memory, favours the higher susceptibility of newborns to infection and intracellular or capsulated pathogens [38]. In addition, antibodies produced by newborns have a shorter duration, are initially produced later, and show lower affinity, reduced heterogeneity, and deficient response to bacterial polysaccharides [27]. Further limiting vaccination after birth, plasma cells show low induction and less migration to the bone marrow, which contributes to limited humoral response and rapid decline of vaccine antibodies [28, 29, 39].

## 4. Vaccination

The introduction of a combined diphtheria/tetanus/whole cell pertussis vaccine (DTwP) in the 1940s and 1990s has been effective in the large decrease in pertussis morbidity and mortality in young children. Between 1999 and 2014, the World Health Organization (WHO) records suggest that more than 100,000 infant deaths could have been prevented mainly by increased coverage of pertussis vaccination [40].

The appearance of adverse reactions such as convulsions and encephalopathies [41] led the development of an acellular pertussis vaccine (aP) based on purified *B. pertussis* antigens, which were less reactogenic than the whole-cell vaccine [42, 43].

Acellular vaccines are presented in two formulations: DTaP, for vaccination of children, and Tdap, for vaccination of adults, with higher or lower concentrations of diphtheria toxoid and pertussis antigens [44]. For the immunization of individuals older than 7 months of age, Tdap is recommended rather than DTaP due to adverse reactions increasing with age and number of doses [45]. Many countries replaced DTwP for acellular vaccines, but countries such as Brazil and Argentina still use the whole-cell formulation since acellular formulations have a much higher cost [45].

Despite the differences between adult and children immune systems, it is known that DTwP in children can prime their immune system for a Th1 response, while acellular vaccines induce a mixed Th1/Th2 response, with production of both IFN-*γ* and IL-4 [46, 47]. There are studies showing that children vaccinated with DTaP in the first year of life produced more IFN-*γ* than IL-5 [47, 48]. However, this relationship shifted after boost doses [47] or natural asymptomatic infections [4].

Regarding antibodies, primary immunization of children with DTwP showed induction of specific anti-PT, anti-FHA, and anti-pertactin IgG, since the first dose at 2 months of age and lasting until 2 years of age [49] or longer [50, 51]. Anti-PT IgG was also positively correlated with protection of children after aP administration [52].

The WHO recommends the administration of three doses in the first year of life to decrease the incidence of pertussis. Vaccination is recommended at six weeks of age with the other two doses administered at 4- and 8-week intervals, respectively, until the child's sixth month of life [45].

The organization states that both cellular and acellular vaccines are safe and effective in disease prevention in the first year of life [45]. However, it recommends that low- and middle-income countries that use DTwP should not replace the vaccine since the whole-cell formulation is low-cost (US$ 0.38 per DTwP dose compared with US$ 9.15 per DTaP dose) and highly effective, without directly related, important adverse events reported [53, 54].

Nevertheless, data from mathematical modelling [55] and baboon experiments [56] show that even though the acellular vaccine is capable of preventing serious symptoms, it does not prevent bacterial colonization. Since DTwP consists of whole bacteria, this vaccine elicits antibodies and cytokines against a wider range of antigens, which may affect attachment to the respiratory tract and bacterial opsonization [57]. Therefore, despite vaccination, animals and people could still transmit the bacteria. This could be a possible explanation for the pathogen permanence in aP-vaccinated countries, as well as occasional mutations that can lead to immune response evasion by the bacteria [58].

It is well known that immunity after natural infection is not permanent, decaying after 4-20 years after infection [59]. Therefore, immunity after vaccination is also not long lasting, but immunity after DTwP lasts from 4 to 12 years, and immunity after aP lasts an even shorter period, even after booster doses [53, 59–61].

Thus, the WHO alerts to the possibility of pertussis reemergence, especially in countries using aP vaccines alone. The WHO suggests the use of booster doses at two years of age, during pregnancy and in people directly in touch with young children to avoid serious pertussis cases in at-risk groups [62], as newborn and infants are the most susceptible groups to disease [45] and infection in these groups results in a higher lethality rate [63]. The main contagious sources are siblings and parents [64–66], and countries such as Australia, Germany, and the US recommend booster doses in these groups [67].

The vaccination scheme against pertussis needs to undergo a reevaluation, especially since there has been a resurgence of this disease. Recent vaccine candidate studies explored live-attenuated formulations that cause lower adverse reactions than DTwP and include possible mutations of the wild-type bacteria with the goal of also reducing transmission of the pathogen [68–70]. One example is a whole-cell vaccine produced with lower endotoxin levels developed by the Butantan Institute, Brazil, aimed at reducing severe reactogenicity caused by the bacteria's lipooligosaccharide [70].

Nevertheless, while new vaccines were in trials, different vaccination strategies were implemented, such as neonatal immunization (from birth to 1 month of age), cocooning, postpartum vaccination, and maternal immunization, to increase protection levels and prevent further pertussis cases [71, 72].

## 5. Prevention Strategies

### 5.1. Vaccination at Birth

The viability of immunizing children soon after birth with DTwP has been studied for more than 40 years but has been discontinued because it results in immunological tolerance, where the levels of antibodies to *B. pertussis* antigens are reduced compared to those in children who were vaccinated later [73].

However, while aP vaccination in neonate mice induced low antibody responses [74], recent studies show that acellular vaccine after birth can elicit specific antibody responses during the neonate phase [72, 75]. Supporting the results found in humans, the study conducted by Warfel and coworkers [76] using a baboon model showed protection of newborns against pathogen challenge.

### 5.2. Cocooning

Cocooning has been recommended since the early 2000s in the US, France, Australia, and Germany to prevent pertussis in newborns [45, 77, 78]. This strategy consists of vaccinating all the close relatives when a child is born [79]; this population should receive Tdap at least two weeks before initiating close contact with the child [80]. Cocooning is mainly directed to reduce disease-associated morbidity and transmission to young, unvaccinated children [81]. Recently, cocooning was recommended in Latin America in Brazil, Chile, and Costa Rica [45]. In Chile, in 2012-2013, it is estimated that 84% of potential pertussis deaths in infants were prevented [82], but in other countries around the world, current data found a minor or no impact of this approach [83]. Therefore, the efficacy of cocooning is limited because the child has no specific protection, and the approach demands the vaccination of several adults, making cocooning both costly and difficult to implement [79]. Cocooning is, however, still practiced and studied in several countries (reviewed by Forsyth et al. [84]).

### 5.3. Postpartum Vaccination

In countries such as Brazil and the US, postpartum vaccination or partial cocooning is recommended as early as possible for women not vaccinated during pregnancy to prevent the mother from transmitting pertussis to the newborn [85, 86], but this strategy is not ideal because it offers protection only to the mother. After vaccination, it takes two weeks to generate a maximal immune response to the vaccine antigens, during which time the mother is vulnerable to contracting and disseminating the disease to the child [86]. It is possible that the postpartum immunization of mothers can be administered too late to protect newborns if the mother is already infected during childbirth or is exposed to pertussis soon after [87].

### 5.4. Maternal Immunization

The most recent strategy is aimed at inducing higher anti-pertussis antibody levels in pregnant women and higher placental transfer rates to the foetus [77], since pertussis has no protection conferred by natural maternal antibodies (MatAbs) [88], and children are most vulnerable to infection during the first months of life because they are not fully vaccinated [77].

Countries such as the US, UK, and Australia have recommended maternal immunization since 2012 for both the protection of children and mothers [89]. Efficacy and security studies have been published in these countries, indicating decreased hospitalization and disease severity [90–94]. In Brazil, the incidence dropped from 4.2/100,000 to 0.9/100,000 cases/inhabitants after introduction of the vaccine [95].

Maternal antibodies are transferred especially during the third trimester of pregnancy, with a half-life in the newborn of approximately six weeks [13]. Before the 16th gestational week (GW), fetal IgG serum levels correspond to 8% of the maternal levels, but the fetal levels increase until they reach maternal levels at 26 GW, especially for IgG1. Neonates reach adult levels of self-produced IgG at 3 years of age [96].

Several studies estimated the best period for vaccination, which was found to be between 27 and 31 weeks of gestation [97–99] when anti-PT IgG has higher affinity. However, other studies show that earlier immunization promotes higher antibody transference to the newborn, most likely due to a cumulative effect, which can be especially important regarding preterm neonate protection [100, 101].

In the US, vaccination is still recommended at the 27th GW and onwards [102]. However, in the UK, women are vaccinated starting in the 16th GW [103], and in Brazil, even though the vaccine was initially administered from the 27th to the 36th GWs, initial administration recently changed to start at the 20th GW to reach a higher number of women [104], and this timing has been proven to be cost-effective [105].

However, since the 1950s, it is known that maternal antibodies can interfere in the child's own immune response to vaccination [106]. There are many studies on this subject, especially in vaccination models of tetanus and measles [107–110].

The interval between immunization and birth can determine the behaviour of antibodies when they contact vaccine antigens in infancy; this behaviour may depend on the vaccine formulation (acellular or whole-cell vaccine) given to the child [27, 107].

High antibody concentrations can suppress the child's immune response by not completely elucidated mechanisms. The most accepted theories state that maternal antibodies can form immune complexes with vaccine antigens, either inhibiting neonatal B lymphocyte activation or eliminating the antigen via antibody-dependent phagocytosis, and can mask antigenic epitopes, preventing antigens from bonding with neonatal B lymphocytes [27, 110]. However, this lack of B-cell stimulation may be compensated by the noninterference in the induction of the T-cell response in the neonate; the complexes formed by MatAbs and bacterial peptides are captured by phagocytes and presented to naïve T cells, allowing them to be activated and to differentiate [33].

Recent humoral studies show lower antibody levels in children born from vaccinated mothers [111, 112], but preliminary studies show that the T cell remains unchanged (Argondizo et al., unpublished data); however, larger and more representative studies must be developed to evaluate possible interactions.

## 6. Discussion and Conclusion

Globally, pertussis reemergence is a challenge in developed and developing countries, with high morbidity and mortality rates in young children [71]. Several strategies (reviewed in Table 1) have been recommended to compensate for incomplete immunization until the sixth month of age [45], but during the first year of life, children are exposed to an environment full of antigenic stimuli and are highly susceptible to infections.

Even though children ultimately develop an immune system that is able to respond to infection, there is a need to initially balance between hypoinflammation and hyperinflammation once the transition from the partially sterile placental environment to the external environment is complete [25]. As pathogen recognition is mediated by the same receptors that recognize commensal organisms that colonize the neonate from birth, these receptors must be regulated to avoid harmful inflammation [25]. Therefore, neonates' immune responses are usually lower and less effective when compared to those in adults. Regular pertussis vaccination in early life can induce protection, but protection only starts at 2 months of age [113]. Some studies have shown that newborn vaccination is well tolerated [73, 114], but there is no direct evidence currently available, and it remains controversial whether newborn vaccination can interfere with future vaccinations.

Vaccinating mothers just after labour and/or close relatives in the cocooning strategy can prevent disease spread, but there are difficulties for large-scale implementation of these approaches because they are costly and require vaccination of multiple people in addition to leaving the children born with no specific protection [79].

Maternal vaccination, on the other hand, provides protection for both the mother and the newborn, and a study on the safety and immunogenicity of vaccination in the third trimester of pregnancy revealed that babies born to vaccinated mothers have higher concentrations of antibodies against pertussis at birth and at two months of age compared to babies born to postpartum, immunized mothers [115]. Nevertheless, maternal antibodies can have inhibitory effects on the child's immune response, and these effects should be further investigated [110].

Despite all existing strategies, no vaccine prevents the silent transmission of the pathogen, so new formulations are needed. Several options are being explored and have shown promise in preclinical studies in the search for new and more efficient live-attenuated vaccines against pertussis [116]. Some of these formulations are based on increasing knowledge that has been accumulated in recent years about immunity against *B. pertussis*, mainly regarding the role of Th1 and Th17 cells in addition to antibodies [68].

Overall, advances in knowledge regarding neonatal immunity and pertussis immunopathogenesis support that vaccination is the best strategy to fight pertussis, and studies concerning different vaccination periods have supported the development of more effective vaccines and strategies to overcome high susceptibility and severity of pertussis in early life.

## Figures and Tables

**Table 1:** Advantages and disadvantages of infant protection strategies.

Strategy	Objective	Number of doses	Advantages	Disadvantages	References
Child vaccination with DTwP	Induce specific protection in children	3 doses in the first year of life and 2 boost doses	(i) Th1 response induction (ii) Antibody response (iii) Prevent pertussis symptoms	(i) Higher risk of local and systemic adverse reactions (ii) Immunity lasts for 4-12 years	[41, 46–49, 53, 59–61]

Child vaccination with DTaP	Induce specific protection in children with less side effects	3 doses in the first year of life and 2 boost doses	(i) Less reatogenic than DTwP (ii) Primes a Th1/Th2 response (iii) Antibody response (iv) Prevent pertussis symptoms	(i) Do not prevent bacterial colonization and transmission (ii) Immunity lasts for a shorter period of time than DTwP	[41–43, 46–49, 52, 53, 55, 56, 59–61]

Mother post-partum vaccination with Tdap	Confer protection to mothers and prevent child contamination	1 dose after labour	(i) Protect the mother to transmit the disease	(i) Confers protection only to the mother, and after two weeks from vaccination	[85–87]

Newborn vaccination	Induce protection in children as soon as they are born avoiding the first two months of age being unprotected	1 dose just after birth	(i) First dose still in the hospital	(i) (wP) Immunological tolerance; lower antibody production (ii) (aP) Antibody production and protection against experimental challenge	[72, 73, 75, 76]

Cocooning	Create a protected environment for unvaccinated children	1 dose for every relative, every time a child is born	(i) Prevent contamination of the unprotected child	(i) Costly (ii) Difficult to implement (iii) Child remains without specific protection	[79, 81, 84]

Vaccination with Tdap during pregnancy	Induce protection in mothers and transmit specific passive protection to the foetus and newborn, until the child's vaccination	1 dose from the 20th to the 36th gestational week, in every pregnancy	(i) Induces specific protection in children (ii) Just one dose for every pregnancy (iii) Cost-effective (iv) T cell responses remains unaffected by maternal antibodies inhibition	(i) High maternal antibody concentration can interfere in the child's immune response	[77, 104–106]
